# Chronic Bumetanide Infusion Alters Young Neuron Morphology in the Dentate Gyrus Without Affecting Contextual Fear Memory

**DOI:** 10.3389/fnins.2020.00514

**Published:** 2020-05-21

**Authors:** Gibrán Gómez-Correa, Angelica Zepeda

**Affiliations:** ^1^Instituto de Investigaciones Biomédicas, Universidad Nacional Autónoma de México, Mexico City, Mexico; ^2^Institute of Clinical Neuroanatomy, Goethe University Frankfurt, Frankfurt, Germany

**Keywords:** neuronal maturation, pediatric epilepsy, diuretics, GABA, neurogenesis, transverse axis

## Abstract

Young neurons in the adult brain are key to some types of learning and memory. They integrate in the dentate gyrus (DG) of the hippocampus contributing to such cognitive processes following timely developmental events. While experimentally impairing GABAergic transmission through the blockade or elimination of the ionic cotransporter NKCC1 leads to alterations in the proper maturation of young neurons, it is still unknown if the *in vivo* administration of common use diuretic drugs that block the cotransporter, alters the development of young hippocampal neurons and affects DG-related functions. In this study, we delivered chronically and intracerebroventricularly the NKCC1 blocker bumetanide to young-adult rats. We analyzed doublecortin density and development parameters (apical dendrite length and angle and dendritic arbor length) in doublecortin positive neurons from different subregions in the DG and evaluated the performance of animals in contextual fear learning and memory. Our results show that in bumetanide-treated subjects, doublecortin density is diminished in the infra and suprapyramidal blades of the DG; the length of primary dendrites is shortened in the infrapyramidal blade and; the growth angle of primary dendrites in the infrapyramidal blade is different from control animals. Behaviorally, treated animals showed the typical learning curve in a contextual fear task, and freezing-time displayed during contextual fear memory was not different from controls. Thus, *in vivo* icv delivery of bumetanide negatively alters DCX density associated to young neurons and its proper development but not to the extent of affecting a DG dependent task as aversive context learning and memory.

## Introduction

The subgranular zone of the rodent dentate gyrus harbors neural progenitors that divide symmetrically or generate intermediate neural progenitors, which in turn can self-renovate or differentiate as neuroblasts. Neuroblasts can divide and while in a neuroblast-like stage, they migrate toward the inner zone of the granular layer of the dentate gyrus where they exit the cell cycle, differentiate into young neurons and continue their maturation and integration process (Kuhn et al., 1996, for a review, see Kempermann et al., 2015). Young neurons represent a relatively low population of cells within the dentate gyrus (6%) but display higher synaptic plasticity and excitability than mature neurons (Cameron and McKay, 2001). Young neurons have been shown to participate in contextual fear conditioning (Saxe et al., 2006; Clelland et al., 2009; Hernández-Rabaza et al., 2009; Tronel et al., 2012), spatial memory (Snyder et al., 2005; Abrous and Wojtowicz, 2015), and cognitive flexibility (Burghardt et al., 2012) while computational approximations that simulate the neurogenic process hint its involvement in an increased capacity to remember new situations (Weisz and Argibay, 2009; for a review see Deng et al., 2010). Also, the capacity of the DG to increase the resolutive capacity to code similar stimuli differently has been suggested to depend on the recruitment of new, young neural cells (Weisz and Argibay, 2009; Satvat et al., 2011). GABA is the main inhibitory neurotransmitter in the adult brain. However, during development of neurons GABA functions as a depolarizing stimulus (Ben-Ari et al., 1989) and in consequence, as a signal that promotes maturation, proliferation, migration and synaptic formation (Ge et al., 2006; for a review, see Palloto and Deprez, 2014). GABAergic tonic and phasic stimuli are excitatory in young cells given that they express the ionic cotransporter NKCC1 whose activity raises intracellular concentration of Cl^–^ (Hollrigel et al., 1998; Ganguly et al., 2001). GABAergic stimulation is particularly important to neuronal maturation (Rivera et al., 1999; Ben-Ari, 2002); as neural progenitors mature, the expression of the NKCC1 cotransporter fades and at the same time there is an increase in the expression of the ionic cotransporter KCC2, which transports K^+^ and Cl^–^ ions outside the cell (Yamada et al., 2004; for a review see Blaesse et al., 2009). The change of the depolarizing effect of GABA to a hyperpolarizing one in young neurons through the silencing of the gene for NKCC1 produces synaptic impairment and halts dendritic development (Ge et al., 2006). These findings pose GABAergic activity as an essential stimulus for the maturation and functionality of young neurons (Ge et al., 2006; Palloto and Deprez, 2014). These observations are of clinical relevance given that the NKCC1 cotransporter is a pharmacological target of furosemide and bumetanide (Walcott et al., 2012; for a review see Ben-Ari and Tyzio, 2011). Bumetanide is a relative specific inhibitor of the NKCC1 cotransporter currently used as a diuretic (Hannaert et al., 2002; Walcott et al., 2012) that blocks GABA-induced depolarization in immature neurons through the inversion of the membrane potential and the GABA reversor potential (Wang and Kriegstein, 2011). While bumetanide has been shown to cause permanent cortical circuit alterations (Wang and Kriegstein, 2011) its use has been suggested for treating pediatric epilepsy and cerebral edema (Dzhala et al., 2005; Walcott et al., 2012). Therefore, given the interest that poses the clinical use of bumetanide, in the present study we analyzed the long-term effect of the intracerebral delivery of bumetanide on the structural maturation of young hippocampal dentate gyrus neurons and evaluated its impact on contextual fear conditioning and memory in rats.

## Materials and Methods

The animal study was reviewed and approved by the ethics committee at the Instituto de Investigaciones Biomédicas, Universidad Nacional Autónoma de Mexico (UNAM) (NOM 062-ZOO-1999). Experiments were conducted with efforts to keep the welfare of the animals and followed the lineages of standard biosecurity and safety procedures as dictated by the Ethics Code of the Instituto de Investigaciones Biomédicas, UNAM.

### Subjects

A total of 14 male Wistar rats (250–350 g) were kept under standard housing conditions with food and water *ad libitum* in an inverted 12 h/12 h light-dark cycle; the lights were turned on at 19:00 h. Behavioral evaluations were conducted during the active phase of the animals. Subjects were divided equally into two groups; the experimental group received bumetanide (Santa Cruz Biotech, United States, Ro 10-6338) diluted in propylene glycol and the control group received the excipient propylene glycol (Sigma Aldrich, United States, W294004) as described below.

### Osmotic Minipump Preparation

Osmotic minipumps (Alzet, Model 2002, approximate capacity of 200 μL, flow rate 0.5 μL/H Durec, Cupertino, CA, United States) were used. The duration of drug delivery for each osmotic minipump was 14 days. Two pumps were used per subject, one replacing the other when the drug delivery-time was over. The pumps for the experimental group were filled with bumetanide dissolved in propylene glycol, as bumetanide fully dissolves in this substance. A concentration of 0.4 mg/kg/day of bumetanide was delivered during 28 days. In average, a solution of 3.92 mg of bumetanide in 200 μL of the excipient was used to fill each osmotic minipump. The pumps for the control group were filled with propylene glycol alone. One night before performing the implant and immediately after the pump was filled, pumping was prompted by immersing the pump in a 0.9% NaCl solution at 37°C overnight.

### Intracerebroventricular Implant of the Osmotic Minipumps

A total of 2% Isoflurane was mixed with 95% O_2_ and 5% CO_2_ to be used as inhalational anesthetic. Animals were mounted in a rat stereotaxic brain unit (Kopf, United States), the scalp area was shaved, the skin was cleaned with an antiseptic solution, and an incision was performed in alignment with the anteroposterior axis over the midline of the skull. The area of the exposed skull was cleaned, and a small unilateral hole was drilled through the skull in the following coordinates from bregma according to Paxinos and Watson (2013): AP −1.4 mm; ML −2.0 mm. A brain infusion kit (Alzet, United States) was used to direct the content of the mini osmotic pump unilaterally to the lateral ventricle of the rat. The infusion cannula was attached to the post of the stereotaxic unit using a holder and the cannula was lowered though the hole in the skull until it reached the coordinate DV 4.0 mm. The base of the infusion cannula was fixed to the skull using dental cement (MDC Dental, United States) and was left untouched until the cement was completely dry. The osmotic mini pump was placed in a subcutaneous pocket formed caudally to the skin incision. The pump was then connected to the infusion cannula through the plastic catheter provided in the brain infusion kit. The skin in the area of the scalp was then sewed together to cover the exposed area and the analgesic lidocaine (7 mg/kg PiSA, México) was administered. After the removal of the inhaled anesthetic, animals were kept under close observation and were then placed in their home cage. Two weeks after performing the implant, animals were anesthetized, and placed in the stereotaxic unit for a pump replacement. The old pump was removed from the skin pocket and was replaced with a fresh pump so that the administration time of either bumetanide or propylene glycol would last 28 days. The catheter connecting the pump to the infusion cannula was clamped while it was disconnected from the old pump and was then reconnected to the new pump. Care was taken that bubbles did not form inside the catheter. The skull implant was left untouched.

#### Habituation

On day 24 after implanting the first minipump and for the two following days, animals underwent 10 min of manipulation by the experimenter in the same room where the behavioral tests would take place.

#### Open Field Test

The test was carried out on day 27 after the first minipump was implanted and was used to evaluate locomotion and as a general measure of anxiety. A black acrylic arena 80 cm long × 80 cm wide × 30 cm tall was used; the floor was divided in 16 equal squares of 20 × 20 cm. The four inner squares were considered the central area of the arena and the remaining 12 outer squares, the periphery. At the beginning of the test, animals were placed on the central area of the arena and left to explore it at will. The room was illuminated with red light; locomotion was recorded for 5 min and the crossings of over each square were registered.

#### Contextual Fear Conditioning

A conditioning chamber 25 cm long × 25 cm wide × 20 cm tall was used (San Diego Instruments, United States). The walls and ceiling of the chamber were made of transparent acrylic and the floor was made of 23 stainless steel bars through which an electric shock (1 mA for 2 s) was administered. The chamber had an array of laser beams that allowed automatic registering of movement inside the cage by sensing the breaking of each laser beam due to movement. During the acquisition phase of the contextual fear task, animals tend to show gradual immobility or “freezing behavior” as the footshocks are delivered. Freezing behavior is defined as the absence of movements except for those necessary for breathing (for a review see Brandão et al., 2008). During the recall phase, animals freeze even in the absence of the aversive stimulus, which is considered to be a measure for the recall of an aversive context. 30 min after subjects performed the open field test, contextual fear conditioning took place. Subjects were individually introduced into the conditioning chamber and were left to explore the chamber freely for 2 min. After this period, we administered five shocks separated by variable intervals in a span of 720 s. Time of immobility or freezing behavior, where no laser-beam breaks occurred, was recorded by the Freeze Monitor program and was compared to the recordings from two independent experimenters, one of which was blind to the grouping of the subjects. Contextual fear memory was evaluated 24 h after conditioning and consisted in placing the animals in the conditioning chamber for 5 min without delivering any footshock. Freezing time or immobility was again recorded.

### Histology and Immunohistochemistry

After 30 min the contextual fear memory task concluded, subjects were sacrificed using a lethal dose of sodium phenobarbital (PiSA, México). Animals were intracardially perfused with a 0.9% NaCl solution followed by an ice-cold solution of 4% paraformaldehyde diluted in phosphate buffer. Brains were extracted and placed in the same fixative solution for 24 h. Fixed brains were first immersed for 24 h in a 15% sucrose solution and were then transferred to a 30% sucrose solution for 48 h. Brains were then frozen at −20°C in a cryostat and 30 μm coronal sections were obtained (Leica Biosystems, Germany).

#### Nissl Histology

**FIGURE 1:**
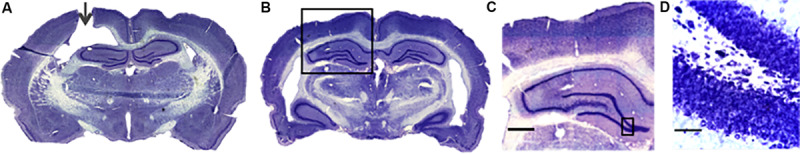
Nissl stained coronal sections from a bumetanide treated animal. **(A)** The micrograph shows a representative section where the infusion cannula (arrow) can be appreciated reaching the lateral ventricle. **(B)** A more posterior section corresponding to the region of the DG where the DCX analysis was performed is shown. The anatomy of the hippocampus is bilaterally similar. **(C)** A 10× magnification of the DG ipsilateral to the infusion shows a regular hippocampal anatomy. **(D)** A 40× magnification shows a regular granular cell layer with no evident pyknotic nuclei. Scale bars: **(C)** 800μm; **(D)** 50 μm.

A series of coronal slices separated by 210 μm were stained with cresyl violet to corroborate the proper placement of the infusion cannula in the right lateral ventricle (Figure 1). At least four sections per animal were analyzed for structural damage or hippocampal malformations due to the infusion.

#### Immunohistochemistry

To identify the presence of young neurons in the hippocampus, brain slices were processed for doublecortin (DCX) immunohistochemistry. First, slices were washed in phosphate buffer solution (PBS, 0.1 M, pH 7.4); the slices were then washed in phosphate buffer saline (0.1 M, pH 7.4) mixed with 2% Triton and 10% normal horse serum for 1 h. Slices were then incubated with the primary antibody goat anti-DCX (Santa Cruz Biotechnology, United States Cat#sc-8066) for 12 h, washed 3 times, 10 min each in PBS and incubated with the secondary antibody Dylight 594 donkey anti-goat (Thermo Fisher Scientific, United States Cat#SA5-10088) for 2 h. Finally, slices were washed in PBS and mounted in glass slides adding DAKO mounting medium for immunofluorescence (Agilent, United States) before placing a coverslip. Mounted sections were kept at 4°C.

#### Dentate Gyrus Image Acquisition

Images from DCX immunoprocessed sections were acquired from the DG ipsi and contralateral to the cannula placement using a confocal microscope Zeiss LSM 5. Four different slices comprising the dorsal DG were analyzed and four non-overlapping field-images per slice comprising the crest as well as the supra and infrapyramidal blades were acquired at high resolution (1024 × 1024 pixel) using a 40× immersion oil objective. Thus, a total of 16 images per DG per subject were analyzed. These images were obtained from the dorsal dentate gyrus within the coordinates: AP: −3.1 to −5.1, taking as reference the atlas from Paxinos and Watson (2013).

#### Analysis of DCX-Density Immunostaining

The tool “Analyze particles” from the program Fiji v.1.52q (Schindelin et al., 2012) was used to analyze the density of DCX+ immunostaining. For image analysis 3–5 different sections per dentage gyrus between −3.1 and −5.1 according to bregma were evaluated; two fields from the supragranular zone, 1 from the crest and 1 form the infrapyramidal zone image were acquired with a resolution of 0.31 μm/pixel using a 40× oil objective (Zeiss). Each optical section in “z” from the acquired fields comprised 2 μm and the analyzed projected *z*-stack thickness in all images was 10 μm. Whole field DCX signal was binarized in each image and density was calculated over an area of 325.67 × 325.67 μm with the same signal detection threshold parameters in all cases; the analysis covered the DCX signal in the whole image and the percentage of signal corresponding to each region (crest, infrapyramidal blade or suprapyramidal blade) was calculated. A further regional analysis was carried on evaluating the DCX signal in the subgranular, the granular and the molecular layers separately for each blade and for the crest. Regional selection parameters were as follows: the subgranular layer was considered to be the first line of cells most proximate to the hilus; the granular layer was considered as the area immediately adjacent to the subgranular layer with observable background noise delimiting the cell bodies of granular cells; and the molecular layer was considered to be the area above the granular layer where processes filled the region and where no background noise delimiting cell bodies could be seen. Delimiting the border between the subgranular and the granular layers has shown to pose difficulties since DCX positive cells outside the subgranular zone lie within the most inner third of the granular cell layer. Each region of interest was manually selected using the “Polygon selection” tool from the FIJI program. A value of density per region from each projected stack was obtained. After obtaining the density values for every stack and their respective regions of interest, values were compared to the total area of the stack (325.67 μm × 325.67 μm) to obtain the percentage of detected DCX signal. Percentages were then rescaled to a range of 0–100%, considering the means from the control group as the maximum value or 100% for further analysis.

The pictures were filtered by setting the particle count to those with a minimal diameter of 2 μm and a range of circularity from 0 to 0.8. “Circularity” is a parameter index that filters out spots or marks from the signal depending on how round they are; in this study, the value of circularity was set at the interval of 0 to 0.8, meaning that those marks over 0.8 circularity, the most round, were left out. This filtering aided clearing the background noise found in the pictures.

Dendritic tracing and dendritic growth analysis. The tool “Simple Neurite Tracer” (Longair et al., 2011) from the program FIJI v.1.52q was used to trace in a semi-automatic manner the dendrites of the DCX+ cells from the stacks described in the previous section. This tracing allowed evaluating the length of individual dendrites starting from the base of the soma toward the tip of each dendrite. The number and length of dendrites from DCX+ cells was analyzed in each image comprising the *z*-stack. All cells selected for analysis had their dendritic tree contained within the section, avoiding reconstructing truncated branches. Primary dendrites were selected as those sprouting directly from the soma; when branching occurred, the primary dendrite was selected as the lengthiest branch. All reconstructed cells had their soma within the subgranular zone and the granular layer; there were no cellular somas found in the molecular layer. No dendrites were left out of the analysis due to their length or reach to an extent of the molecular layer.

Dendritic growth angle analysis was performed using the same tool described above after superimposing a drawing a line following the axis parallel to the subgranular layer in the DG and then tracing each dendrite. Growth angles were calculated by using the “angle tool” and designating the medial side of the images as the initial opening of the angle, thus having an angle of 0° is the same as a flat line parallel to the granule cell layer; this approach is very similar to the one used in a previous study done by Naylor et al. (2008). The only difference was their designation of the 0° angle as the straight line that is normally formed by the apical dendrites as they grow perpendicularly through the granular zone toward the formation of the perforant pathway (Naylor et al., 2008).

#### Statistical Analysis

The results were analyzed using the program Prism v4.2. All statistical tests performed were *t*-Student tests comparing control vs. bumetanide groups in every variable after confirming normality of the data with the Shapiro–Wilk normality test and equal variance with the *F*-test, unless otherwise stated. A Mann-Whitney test was also performed if the data failed to pass the Shapiro–Wilk normality test.

## Results

### Histological Analysis

We performed Nissl staining from the extracted brains to corroborate the positioning of the infusion cannula. Figure 1 shows a representative micrograph from a coronal section of a bumetanide treated animal. Figure 1A shows the cannula tract reaching the lateral ventricle. Figures 1B–D shows that no structural damage in the hippocampus (i.e., pyknotic nuclei or structural displacement) was observed.

### Density of Doublecortin (DCX+ Immunofluorescence and Morphology of DCX+ Cells

We analyzed the impact of bumetanide or the excipient propylene glycol on the density of DCX immunostaining as a marker of young neurons and evaluated some characteristics of young neuronal maturation such as number of developed primary dendrites, as well as length and angle of growth of the primary dendrites in the dorsal DG. Density analysis was performed separately for: the crest, the infra and the suprapyramidal layers. A further analysis was performed for the subgranular zone, the granular and the molecular layers in each of the blades and in the crest. Analysis was performed in images obtained from both hemispheres (ipsi and contralateral to the cannula) and data were pooled after a two-way ANOVA followed by a Sidak’s multiple comparison tests revealed no significant differences between hemispheres in the different analyzed regions. Results show no differences in DCX density between the ipsilateral and contralateral sides within the control or bumetanide groups and show a consistent difference between treatment groups when compared either within the ipsilateral or contralateral sides for the Infrapyramidal blade (two-way ANOVA, Interaction: *p* = 0.792, Side: *p* = 0.792, Treatment: *p* = 0.005; Sidak’s multiple comparison test, ipsilateral: *df* =20, *p* = 0.115, contralateral: *df* = 20, *p* = 0.044); and for the Suprapyramidal blade (two-way ANOVA, Interaction: *p* = 0.832, Side: *p* = 0.832, Treatment: *p* = 0.007; Sidak’s multiple comparison test, Ipsilateral: *df* = 19, *p* = 0.1318, Contralateral: *df* = 19, *p* = 0.059); but not for the Crest (two-way ANOVA, Interaction: *p* = 0.957, Side: *p* = 0.957, Treatment: *p* = 0.058; Sidak’s multiple comparison test, Ipsilateral: *df* = 19, *p* = 0.290, Contralateral: *df* = 19, *p* = 0.330).

**FIGURE 2:**
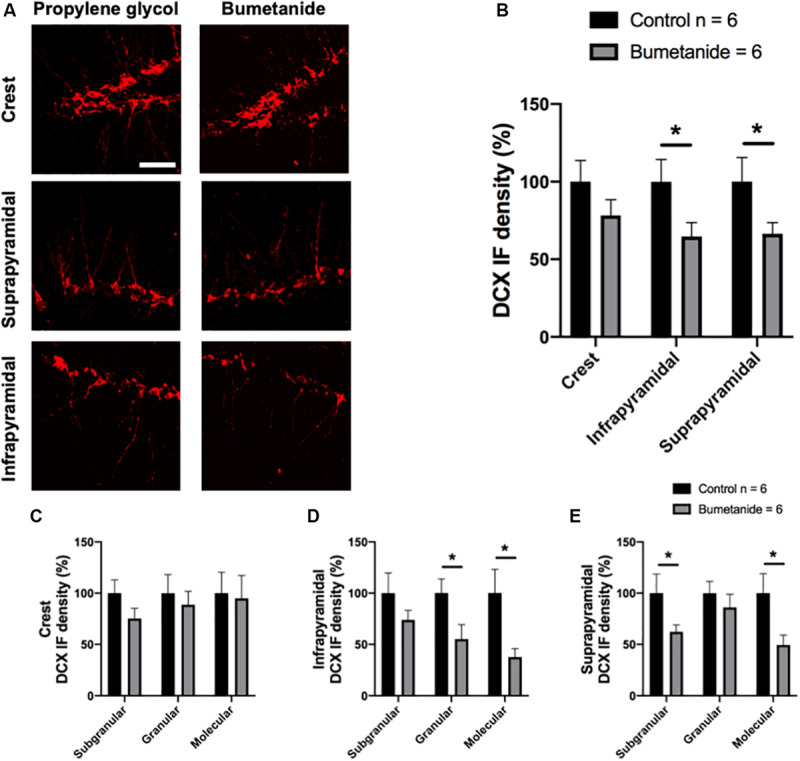
Representative images and immunofluorescence (IF) density analysis of doublecortin in the dentate gyrus of bumetanide and propylene glycol treated animals. **(A)** The left panel corresponds to the control group treated with propylene glycol and the right panel, to the experimental group treated with bumetanide. Upper: Crest, middle: suprapyramidal blade, lower: infrapyramidal blade. Each image is a *z*-stack of 8–10 images. **(B)** The graph shows the doublecortin (DCX) IF density from binarized images in the three different analyzed regions: crest, infra and suprapyramidal blade. A statistical reduction in DCX density IF was found in the bumetanide group compared to the same areas in the control group for the infrapyramidal blade [*t*(10) = 2.08, *p* = 0.031] and for the suprapyramidal blade [*t*(10) = 1.95, *p* = 0.039] but not for the crest [*t*(10) = 1.27, *p* = 0.115); **(C–E)** Graphs show the doublecortin (DCX) density IF from binarized images in the subgranular zone, granular layer and molecular layer from the crest **(C)**, the infrapyramidal blade **(D)** and the suprapyramidal blade **(E)**. The bumetanide group shows a statistical reduction in DCX density IF in the granular [*t*(10) = 2.26, *p* = 0.023] and molecular layers [*t* = 2.53, *p* = 0.014] from the infrapyramidal blade and in the subgranular [*t*(10) = 1.89, *p* = 0.043] and molecular layers [*t*(10) = 2.36, *P* = 0.019] from the suprapyramidal blade; *t*-student. Mean ± SEM per group based on data obtained in 3–4 sections per subject; 2–3 *z*-stacks per field and 8–10 images per stack. Scale bar: 50 μm. *p < 0.05.

Figure 2A shows representative images from the different regions where DCX density was analyzed in both groups. A *t*-student test of the percentage of DCX density in the crest, infra and suprapyramidal blades comparing treatments, revealed a significant reduction in the infrapyramidal blade [*t*(10) = 2.08, *p* = 0.031] and in the suprapyramidal blade [*t*(10) = 1.95, *p* = 0.039] in the bumetanide group compared to the same areas in the control group. No significant differences were observed between bumetanide and control groups in the crest [*t*(10) = 1.27, *p* = 0.115] (Figure 2B).

A *t*-student test of the percentage of DCX density in the subgranular, granular and molecular layers also revealed differences between control and bumetanide infused animals for the infra and suprapyramidal layers but not for the crest when bumetanide infused animals were compared to controls (Figures 2C–E). For the crest no significant differences were found in the subgranular [*t*(10) = 1.50, *p* = 0.081], granular [*t*(10) = 0.50, *p* = 0.313], or molecular layers [*t*(10)= 0.16, *p* = 0.435] (Figure 2C). For the infrapyramidal blade, a significant reduction in DCX density was observed in the granular [*t*(10) = 2.26, *p* = 0.023] and molecular layers (*t* = 2.53, *p* = 0.014) and a non-significant, but clear lower trend was observed for the subgranular layer [*t*(10) = 1.20, *p* = 0.128] (Figure 2D). For the suprapyramidal blade a significant reduction in DCX density was observed in the subgranular [*t*(10) = 1.89, *p* = 0.043] and molecular layers [*t*(10) = 2.36, *P* = 0.019] but not for the granular layer [*t*(10) = 0.80, *p* = 0.220] (Figure 2E).

### Number and Length of Dendrites in DCX+ Cells

**FIGURE 3:**
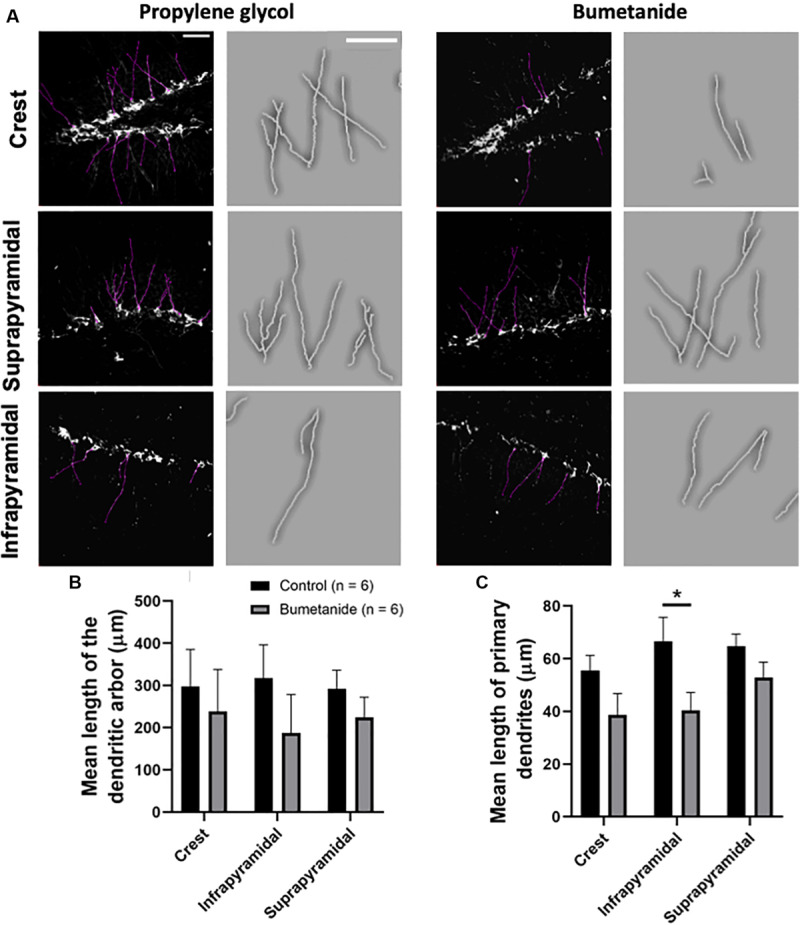
Dendritic skeletons from doublecortin positive cells in the dentate gyrus of bumetanide and propylene glycol treated animals. **(A)** Black and white images show z-stacks where observed dendrites were delineated (purple lines). A magnified manual tracing of a set of dendrites displayed in each black and white image is shown to the right of each image; dendritic tracings are shown in white. Images correspond to the crest (top); the suprapyramidal blade (middle) and the infrapyramidal blade (bottom). **(B)** Mean lengths of the dendritic arbor show no statistical differences between groups in any analyzed region (see text). **(C)** Mean lengths of the primary dendrites in the bumetanide treated group are significantly decreased in the infrapyramidal blade [*t*(10) = 2.31, *p* = 0.021] but not in the crest [*t*(10) = 1.69, *p* = 0.060 nor in the suprapyramidal blade *t*(10) = 1.58, *p* = 0.072] compared to the propylene glycol treated group. *t*-student. Mean ± SEM. Scale bars: 50 μm. *p < 0.05.

Young cells extend their dendrites as a result of their maturation process (Plümpe et al., 2006). In regards to the length of the dendritic arbor, our results show no statistical differences between groups when comparing the suprapyramidal blade [*t*(10) = 1.02, *p* = 0.163], the infrapyramidal blade [*t*(10) = 1.07, *p* = 0.153] or the crest [*t*(10) = 0.45, *p* = 0.330]; since the normality test failed only for the bumetanide group regarding the crest and the infrapyramidal blade, Mann-Whitney tests were also performed resulting in consistent non-statistical differences between groups when comparing, the crest (*U* = 11; *p* = =0.154) or the infrapyramidal blade (*U* = 13; *p* = =0.242) (Figures 3A–C). We also analyzed the length of apical primary dendrites evaluating separately the crest, the suprapyramidal and the infrapyramidal blades of the dorsal granular cell layer. Our results show that the length of apical primary dendrites in the crest and suprapyramidal blade showed no significant differences [*t*(10) = 1.69, *p* = 0.060; and *t*(10) = 1.58, *p* = 0.072, respectively], while the length in the infrapyramidal blade was statistically reduced in the bumetanide group when compared to the propylene glycol group [*t*(10) = 2.31, *p* = 0.021] (Figure 3C).

### Angle of Growth of Primary Dendrites

**FIGURE 4:**
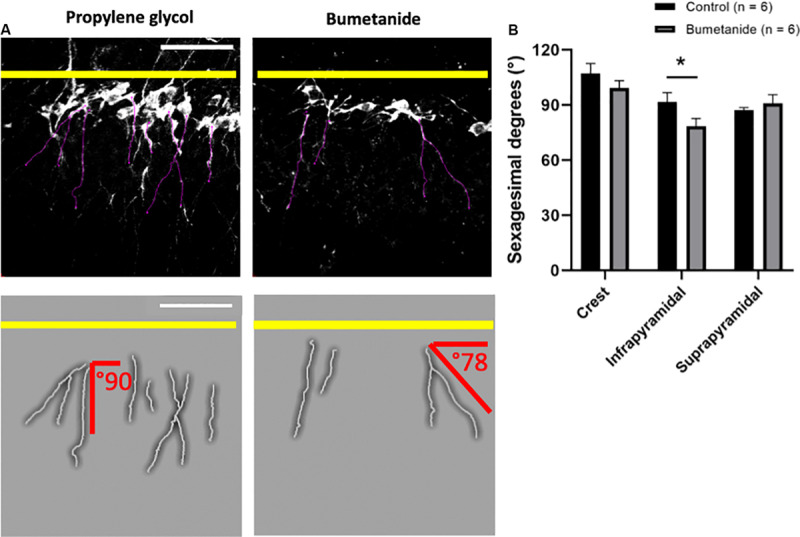
Dendritic growth angles in primary apical dendrites. **(A)** Representative images from doublecortin positive cells in the infrapyramidal layer. Black and white images show *z*-stacks from where dendrites were delineated (purple lines). A magnified manual tracing of a set of dendrites displayed in each black and white image is shown below each image; dendritic tracings are shown in gray and degrees of growth of two dendrites with respect to the granular zone (marked with a yellow line) are depicted in red. Notice that growth angles are predominantly straight in the control group and slanted in the bumetanide group. **(B)** Mean sexagesimal degrees from primary apical dendrites in the bumetanide treated group are significantly different from the control group in the infrapyramidal blade [*t*(10) = 2.03, *p* = 0.034], but not in the crest nor the suprapyramidal blade [t(10) = 1.15, *p* = 0.137; and *t*(10) = 0.73, *p* = 0.238, respectively]. *t*-student; mean ± SEM. Scale bars: 50 μm. *p < 0.05.

The analysis of the orientation or angle of growth of the DCX+ apical primary dendrites revealed significant differences between treatments only for the infrapyramidal blade [*t*(10) = 2.03, *p* = 0.034]. The crest and the suprapyramidal blade did not show significant differences [*t*(10) = 1.15, *p* = 0.137; and *t*(10) = 0.73, *p* = 0.238, respectively] (Figures 4A,B).

### Behavioral Analysis: Open Field, Contextual Fear Conditioning and Contextual Fear Memory

**FIGURE 5:**
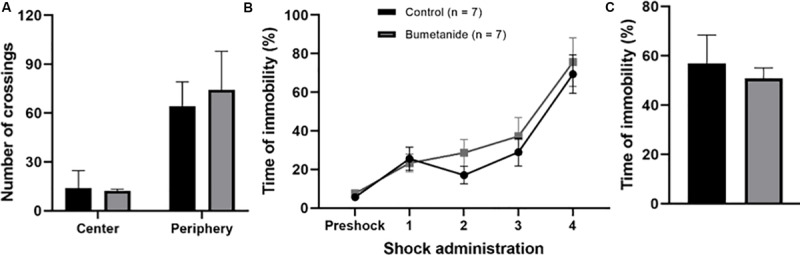
Performance in the open field and in contextual fear learning and memory. **(A)** Bars show the mean crossings for central and peripheral squares. There were no differences between groups for the preference to enter central [*t*(12) = 0.41, *p* = 0.690] or peripheral areas [*t*(12) = 0.95, *p* = 0.360]. Mean ± SEM; *t*-test. **(B)** Contextual fear memory learning curve in a 12 min session. Shocks were randomly delivered after two min of introducing the animal in the chamber. During conditioning both groups reached a maximum of 70% immobility time showing that treatment did not affect their learning capacity. No significant differences along the learning process were observed. **(C)** Contextual fear memory. Time of immobility was similarly high between groups during the recall session showing that animals from both groups remembered the aversive context after 24 h of learning [*t*(12) = 0.26, *p* = 0.318). Mean ± SEM; *t*-test.

With the aim of evaluating if DCX density as well as if modifications observed in DCX positive neurons associated to bumetanide treatment had an impact upon a DG dependent task, we analyzed contextual fear memory learning and memory in both treated groups. In addition, we used the open field test to analyze overall anxiety and to control for general mobility (Figure 5). The results show that there were no differences between groups regarding the number of crossings to the central area [*t*(12) = 0.41, *p* = 0.690] or to the periphery [*t*(12) = 0.95, *p* = 0.360). Subjects spent more time in the periphery than in the center of the field, which is expected for rodent behavior (Figure 5A).

Contextual fear conditioning was successfully established in bumetanide as well as in propylene glycol infused groups as shown by their freezing curves (Figure 5B). In both groups, the curve followed a typical increase in freezing time along the 12 min conditioning session reaching a 70% of freezing time after the last shock (Figure 5B). Contextual aversive memory, evaluated 24 h after conditioning, showed that animals displayed the classical initial exploratory behavior followed by immobility or freezing behavior during the 5 min session thus revealing the maintenance of the aversive memory. No significant differences were observed between groups [*t*(12) = 0.26, *p* = 0.318] (Figure 5C).

## Discussion

The dentate gyrus of the hippocampus processes information that ultimately leads to the formation of episodic memories, while specific functions such as contextual memory and pattern separation are highly dependent on the participation of young neurons (Squire, 1992; Nilsson et al., 1999; Clelland et al., 2009; Huckleberry et al., 2018). It is well documented that the blockade of the NKCC1 cotransporter activity impairs tonic GABAergic stimulation during the development of immature neurons and that GABAergic stimulation mediated by NKCC1 physiological activity is a trophic factor for developing neurons (LoTurco et al., 1995). Several studies have shown that cognitive deficits occur when the birth of new neurons is severely impaired (Jaako-Movits et al., 2005; Mohapel et al., 2005; Saxe et al., 2006; Bonnet et al., 2008; Hernández-Rabaza et al., 2009) while eliminating GABA-mediated depolarization in new adult hippocampal neurons has been shown to affect their morphology as well as their synaptic integration (Ge et al., 2006, 2008; Wang and Kriegstein, 2011). However, to our knowledge, the impact of altering the maturation of young DG neurons and the consequences of such alteration in DG-mediated cognitive functions in control conditions had not been evaluated. This gains relevance as common use diuretic drugs such as bumetanide have been shown to interfere with neuronal maturation while clinical trials have been undertaken for its use in treating pediatric epilepsy (Dzhala et al., 2005).

In this work, we analyzed the effects of delivering icv the NKCC1 blocker bumetanide to young-adult rats. For addressing our experimental questions it was paramount that bumetanide reached brain cells and in physiological conditions, systemic administration of bumetanide crosses the blood brain barrier only in a limited fashion (Römermann et al., 2017, for a review, see Kharod et al., 2019). Also, even when this study is not intended to extrapolate the results to the clinics, it should be considered that bumetanide has been suggested to treat brain edema, ischemia and seizures which suppose a disruption of the blood brain barrier. Thus importantly, under pathological conditions, bumetanide may reach brain tissue. The direct delivery of bumetanide to the brain at a constant concentration allowed us to reproduce previous *in vitro* observations showing that altering GABAergic transmission negatively impacted on the morphological development of young neurons. The disadvantage of this experimental method is that it is invasive and the tract of the cannula produces some mechanical damage at the cortical level. It should be, however, mentioned that in our hands, this did not seem to lead to behavioral deficits, nor to damage (i.e., pyknotic nuclei indicating death, or structural displacement) beyond that generated by the tract (see Figure 1).

We tried several concentrations and durations of bumetanide treatment (data not shown) and conducted the experiments with the conditions that clearly showed an impact on the morphology of DCX+ cells to then analyze if these alterations had an impact on contextual fear learning and memory, which have been shown to depend to some extent, in young neurons. Our rational was that if blocking GABAergic transmission affected young neurons, as has been shown to be the case *in vitro* (Ge et al., 2006) and in cortical cells *in vivo* (Cancedda et al., 2007; Wang and Kriegstein, 2011) then, the alterations in the maturation of new neurons could impact DG-related functions in which young cells participate. The latter relates to previous results showing that young new neurons are preferentially recruited over mature granule cells. So, even when they account for a small population, they can contribute to the network function (Kee et al., 2007; Snyder et al., 2009).

### Reduction in DCX Density and Morphological Alterations of Young Neurons

Our results show that intracerebroventricularly administered bumetanide, diminishes DCX-density signal associated to young cells in the DG and alters their morphology (i.e., primary dendritic length and growth angle of the primary dendritic length) in a region-dependent manner. Bumetanide infusion led to a reduced density of DCX immunostaining both in the supra and infrapyramidal blades, reflecting a similar effect in the transverse axis and hence, an overall effect in the DG.

The decreased density of doublecortin may be related to a diminishment in the survival of new neurons due to a disturbed excitatory-inhibitory balance (Wang and Kriegstein, 2011) but may also reflect a lower survival rate of new born neurons in the absence of the trophic stimulus (Overstreet-Wadiche et al., 2006; see Dieni et al., 2013) as well as an impaired morphological development due to decreased protein synthesis. To gain further insight into the effects of bumetanide on young DG cells, we performed a region specific analysis of DCX density in the subgranular, granular and molecular layers. The subgranular zone contains the soma and horizontal processes of the newest neurons; the granular layer contains young DCX+ neurons and their dendrites as well as DCX+ somata (migrating neuroblasts) located mainly in the most inner zone of the granular cell layer (apposed to the subgranular zone); and the molecular layer contains the dendrites of DCX+ cells. Our results show an overall decrease in DCX density in the bumetanide group, while significant effects were observed in the granular and molecular layer for the infrapyramidal blade and in the subgranular and molecular layer for the suprapyramidal blade. Notably, the density of DCX signal in the molecular layer from bumetanide treated animals was significantly reduced in both, the supra and infragranular layers. Thus the diminished DCX signal in the molecular layer may reflect: (i) the long-term duration effects of bumetanide in the development of older DCX+ cells; (ii) the long-term duration effects of bumetanide in the density of DCX positive cells (found to be reduced in the subgranular zone and in the granular cell layer after 28 days of treatment) meaning that a reduction in the number of cells would translate into less dendrites reaching the molecular layer; and; (iii) lower contents of DCX in dendrites.

We also observed an overall regional reduction of dendritic length, including the dendritic arbor and observed that the mean length of the primary dendrite was significantly reduced only in the infrapyramidal layer (Figures 3B,C).

Our observations are in agreement with previous studies, which have revealed that interfering with NKCC1 signaling through its silencing or through bumetanide treatment in non-injured animals, leads to cortical fewer primary and secondary dendrites as well as to decreased dendritic length, dendritic volume, branch levels, branch points, number of dendrite segments and terminal points (Cancedda et al., 2007; Wang and Kriegstein, 2008, 2011). Results from the present work show that the impact of the treatment in the morphology of the cells was different between the supra and infrapyramidal blades: the growth angle of the primary dendrites showed to be significantly different for the bumetanide group only for cells in the infrapyramidal blade. It has been previously shown that the growth orientation of apical dendrites of young DCX+ cells born after irradiation is altered, which may compromise their structural integration and therefore their proper function (Naylor et al., 2008). Thus, by affecting the proper growth angle of a subset of DG neurons, mainly those in the infrapyramidal blade, bumetanide may interfere with their integration. In hand with this observation the length of the primary dendrites was significantly diminished in the infra, but not in the suprapyramidal blade. These differences may reflect the previously described variances along the transverse axis of the DG, where morphofunctional differences between the supra and infrapyramidal blades have been documented (Desmond and Levy, 1985; reviewed in Amaral et al., 2007; Jinno, 2011; Gallitano et al., 2016).

Granular cells in the suprapyramidal blade have also been shown to have more spines than the infrapyramidal blade and therefore the excitatory synaptic input that the suprapyramidal blade receives is higher than the one in the infrapyramidal blade (Desmond and Levy, 1985).

### Contextual Fear Conditioning and Memory Remain Intact

Bumetanide infusion significantly diminished the density of DCX immunofluorescence associated to young cells and altered the morphology of DCX-positive cells in both the supra and infrapyramidal blades. This event, however, was not sufficient to alter contextual fear memory at the studied time point. Experiments analyzing contextual fear memory have often yielded inconsistent results. Several experimental design factors including time before shock onset as well as number of stimuli used for conditioning, among others have been shown to influence behavioral outcome in intact animals. As for time before shock onset it has been shown that animals need time to form a representation of the context that may be further recognized and that when the shock is delivered immediately after the animal is placed in the conditioning chamber, freezing fails to occur (Drew et al., 2010; Maren et al., 2013). In the present study, we included such pre-shock time and conditioning clearly occurred (Figure 5B). Another source of discrepancy in the literature arises from the number of shocks used for conditioning. Poulos et al. (2016) showed that in intact rats, there is a directly proportional relation between delivered number of shocks for conditioning and freezing time in the memory test: in a short-term evaluation scheme of memory (24 h post training) a five shock training protocol in rats led to significantly increased freezing-time compared to that displayed by animals that received one shock. In line with this observation, it has been shown that neurogenesis ablation in mice that received a single, but not multiple shocks, leads to contextual fear memory impairment (Drew et al., 2010; Denny et al., 2012; Tronel et al., 2012; Huckleberry et al., 2018). In rats, however, it has been shown that contextual fear memory is also impaired in ablated animals that received multiple conditioning shocks (Winocur et al., 2006; Saxe et al., 2006). These differences may reflect that in mice, mature neurons have a greater contribution to the contextual fear function than in rats and that the recruitment of mature neurons in a multiple shock paradigm may account for the displayed freezing behavior. Additionally, it has been suggested that multiple shocks involve extrahippocampal structures for contextual fear memory (Drew et al., 2010), which could also account for the lack of effects observed in our study.

It is also important to consider that studies evaluating the role of adult-born neurons in contextual fear memory, have shown that 4 weeks or 6 weeks, but not 2 weeks nor 8 weeks old adult-born neurons contribute to contextual fear memory (Denny et al., 2012; Gu et al., 2012; Huckleberry et al., 2018). Adult-born neurons functionally integrate within the first 4 weeks after birth and a deficit in their integration process may lead to behavioral impairments. In the case of our experimental design, cells had been exposed to bumetanide 4 weeks by the time we evaluated behavior and their integration may have been hampered. Nevertheless contextual fear memory was not impaired. Beyond the conclusion that the impact of bumetanide on young cells was not severe enough as to provoke a deficit, it should be considered that our multiple shock paradigm may have yielded a “ceiling” effect (Poulos et al., 2016) that provoked a DG response beyond the contribution of young neurons, which in turn masked the effects of bumetanide upon this neuronal population. Therefore in future experiments it would be worth evaluating a single shock paradigm as well as a function like pattern separation, which is highly dependent on neurogenesis in the DG (Clelland et al., 2009; Sahay et al., 2011) and which has been shown to be affected in ablated mice, in spite of the preservation of contextual fear memory (Tronel et al., 2012).

Bumetanide-treated subjects displayed normal levels of anxiety and gross locomotion was not impaired as revealed by the results in the open field task. In line with our anxiety-related observations, Wang and Kriegstein (2011) showed that bumetanide treated animals display a lower startle response. However, they also report impairment in sensorimotor gating and other motor behaviors along morphological modifications in cortical neurons. Thus, the drug does not seem to affect emotional states, which have been shown to be modulated by young cells in the ventral DG (Santarelli et al., 2003; Banasr et al., 2006). In the present study we did not analyze the effects of bumetanide in the dorsoventral axis and rather focused on a dorsal cognitive DG-mediated function.

Although we did not evaluate the electrophysiological properties of the affected neurons, our results are consistent with those showing that GABA-mediated depolarization is crucial for proper morphological maturation (Ge et al., 2006; Wang and Kriegstein, 2008) and that the use of drugs that hamper such depolarization may impact the overall development and synaptic function of new neurons born in the adult DG. Therefore, it would remain to be analyzed if the decrease in DCX density, as well as the morphological alterations observed in DCX positive neurons, affect the synaptic features and integration of DG born neurons in the long term and if affected cells survive for longer periods.

In sum, we hereby provide evidence that bumetanide directly delivered to the healthy brain parenchyma alters developmental parameters and density of doublecortin signal associated to young neurons in the DG of young-adult animals but that at the analyzed time-point, these alterations are not sufficient to impact on multiple shock contextual fear memory.

## Data Availability Statement

The datasets generated for this study are available on request to the corresponding author.

## Ethics Statement

The animal study was reviewed and approved by the ethics committee at the Instituto de Investigaciones Biomédicas, Universidad Nacional Autónoma de México (NOM 062-ZOO-1999).

## Author Contributions

GG-C designed the experiments, performed the experiments and the statistical analysis, collected data, and wrote the manuscript. AZ conceived the idea, designed the experiments, discussed the data analysis, supervised the experiments, provided funding, and wrote the final version of the manuscript.

## Conflict of Interest

The authors declare that the research was conducted in the absence of any commercial or financial relationships that could be construed as a potential conflict of interest.
